# Primary hypothalamic lymphoma with clinical findings mimicking pituitary apoplexy: a case report

**DOI:** 10.1186/s13256-021-02866-7

**Published:** 2021-05-25

**Authors:** Mehmet Sözen, Büşra Yaprak Bayrak, Alev Selek, Zeynep Cantürk, Berrin Çetinarslan, Emre Gezer

**Affiliations:** 1 Department of Endocrinology and Metabolism, Kocaeli University College of Medicine, Kocaeli, 41000 Turkey; 2 Department of Pathology, Kocaeli University College of Medicine, Kocaeli, Turkey

**Keywords:** Hypothalamic lymphoma, Hypopituitarism, Diabetes insipidus, Empty sella

## Abstract

**Background:**

Primary central nervous system lymphoma (PCNSL) is a rare but well-known extra-nodal lymphoma, which usually presents with non-Hodgkin B-cell lymphomas. PCNSL is generally located around the ventricle and is often detected as multiple lesions. It is rarely seen in the area of the hypothalamus.

**Case presentation:**

We report the case of a 48-year-old Caucasian woman with progressive short-term memory deterioration, headache, mental confusion, diabetes insipidus (DI) and hypopituitarism. Early findings were suggestive of a pituitary apoplexy. The results of tests performed during the initial admission at the tertiary health center revealed hypernatremia, hypopituitarism and DI. Intravenous hydrocortisone treatment was initiated for the secondary adrenal insufficiency, and 75 mcg/day of levothyroxine was started for the secondary hypothyroidism on the fourth day following hydrocortisone treatment. A daily dose of 120 mg desmopressin melt tablet was started twice a day for polyuria/polydipsia after the patient’s volume status was balanced. A brain magnetic resonance imaging scan revealed a mass lesion in the hypothalamic area, which was surrounded by marked edema. Anti-edema treatment was initially started considering the suggestion by our neurosurgery team. The patient’s clinical and laboratory findings improved after the initiation of the anti-edema therapy. Afterwards, a biopsy was performed, which diagnosed a malignant diffuse large B-cell lymphoma. Subsequently, intravenous high-dose methotrexate-based therapy was started; however, after the second cycle of chemotherapy, the patient died due to sepsis.

**Conclusion:**

In this report, we present a case of hypopituitarism that developed due to the mass effect of hypothalamic lymphoma with clinical findings of pituitary apoplexy. Intracranial masses may cause obvious endocrinological findings related to hypopituitarism, while vague findings may also be observed due to partial failure. Therefore, it is important to perform a comprehensive endocrinological examination at the time of diagnosis in patients with intracranial masses.

## Background

Central nervous system (CNS) lymphoma is a rare disease, constituting less than 3% of all intracranial tumors. The disease occurs in various anatomical regions including the basal ganglia, thalamus, corpus callosum and periventricular white matter, while hypothalamic involvement is rare [1]. Ninety percent of cases are diagnosed as non-Hodgkin B-cell lymphomas, whereas T-cell lymphomas are less commonly seen [2]. Lesions are generally solitary (65%), but multiple lesions may also be seen (35%). It may be challenging to distinguish primary malignant lesions of the hypothalamic-pituitary (HP) region from metastases and infectious or inflammatory diseases. Infiltrative and infectious diseases include tuberculosis, sarcoidosis and Langerhans cell histiocytosis [3]. Clinical findings in CNS lymphomas vary depending on the location and size of the tumor. Endocrine abnormalities are common with hypothalamic lymphoma, and the most common clinical finding is pituitary hypofunction. A number of patients have symptoms of hypothalamic dysfunction as well as pituitary hypofunction [4]. In this article, we present a case of hypopituitarism due to the mass effect of hypothalamic lymphoma mimicking the clinical findings of pituitary apoplexy.

## Case presentation

A 48-year-old Caucasian female patient presented with an increase in headaches, drowsiness, weakness, fatigue, hallucination and polydipsia with a few hypoglycemic episodes in the 15 days prior to the admission. The patient’s personal and medical histories revealed no chronic disease or any chronic treatment, with no history of smoking or alcohol use. The patient was a housewife, had three children and had not worked in any job before. Her mother had diabetes mellitus and coronary heart disease, and her father had diabetes mellitus. At physical examination her weight was 87 kg, height 1.63 m, body mass index 32.7 kg/m^2^, pulse 117 beats per minute, blood pressure 90/60 mmHg and body temperature 35.9 °C. She had a regular heart rate and rhythm with no murmurs, rubs, or gallops on cardiac auscultation. Respiratory system examination was normal, and respiratory rate was 16/ breaths per minute. In the neurological examination, the place and person orientations were preserved, while she was confused and somnolent, with impaired time orientation. There was no sign of any cranial nerve involvement or motor deficit. Muscle strength and deep tendon reflexes were normal. Other physical examination findings showed no pathological abnormality.

**Fig. 1:**
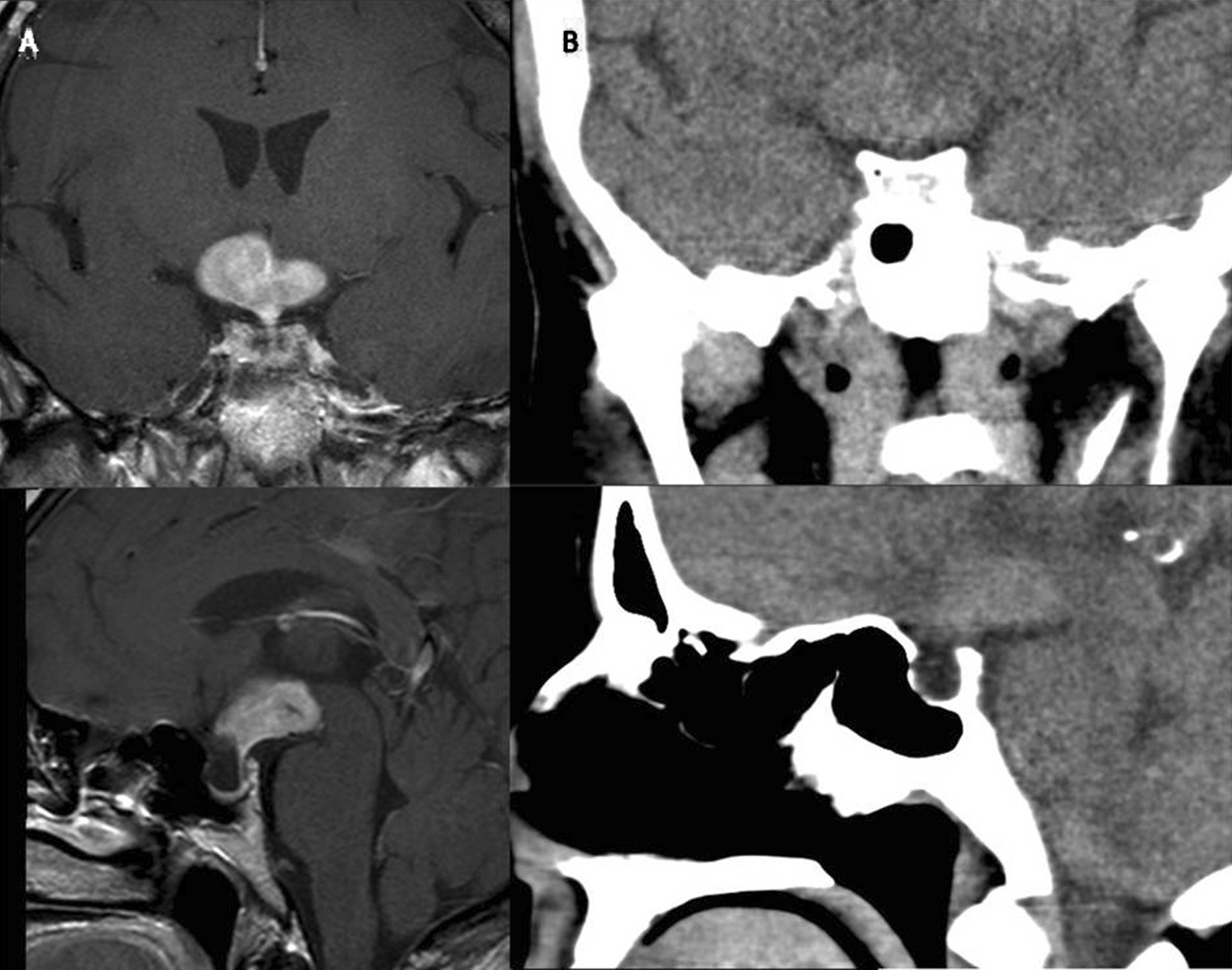
**a** Preoperative contrast-enhanced T1-weighted coronal and sagittal images illustrate a mass originating from the hypothalamus. The infundibulum is fully protected, which is well contrasted and not properly bounded. **b** Non-contrast paranasal computed tomography images showing a hyperdense suprasellar mass and empty sella

Initial biochemical tests indicated hypernatremia, hypopituitarism and diabetes insipidus (DI) (Table 1). Early findings were suggestive of a pituitary apoplexy. Cranial magnetic resonance imaging (MRI) revealed a homogeneously contrast-enhanced mass lesion measuring 22 × 15 × 27 mm. Additionally, diffusion restriction was observed in the hypothalamus, which was surrounded by marked edema. In the differential diagnosis, lymphoma, sarcoidosis and germinoma were considered. Moreover, the pituitary gland volume was decreased, which indicated a partial empty sella (Fig. 1). The angiotensin-converting enzyme (ACE) level was evaluated for sarcoidosis, and was found to be normal. No pathological lymph adenopathy existed in the neck, thorax, abdomen or inguinal regions on radiological images. The patient did not harbor any B symptoms (fever, night sweats, weight loss). The peripheral blood smear examination was normal. Hydrocortisone 10 mg orally every 8 hours was started, followed by a daily oral dose of levothyroxine 75 µg, which was initiated on the fourth day of hydrocortisone treatment. The water deprivation test could not be performed due to the impaired clinical status of the patient. Desmopressin melt tablet 120 mg twice per day was started after the patient’s volume status was balanced. In addition, 75 mL of 20% mannitol four times per day, 10 mg of furosemide every 6 hours, and dexamethasone 8 mg/day in four divided doses were started for the brain edema, considering the suggestion by our neurosurgery team. The general well-being of the patient improved within 4 days of anti-edema treatment. The patient’s clinical symptoms regressed, with the reappearance of confusion, somnolence, polyuria and polydipsia. No episodes of hypoglycemia occurred after treatment.

**Table 1:** Laboratory values observed at initial consultation

Component	Result	Reference range
Hemoglobin	14.2	12.5–16.3 g/dL
Leukocyte	12,500	3.6–10.2/mm^3^
Sodium (Na)	149	136–146 mmol/L
Potassium (K)	4.55	3.5–5.1 mmol/L
Creatinine	0.86	0.51–0.95 mg/dL
Blood urea nitrogen (BUN)	12	7–20 mg/dL
Calcium (Ca)	9	8.8–10.6 mg/dL
Lactate dehydrogenase (LDH)	302	< 248 U/L
Sedimentation	9	< 25 mm/h
Adrenocorticotropic hormone (ACTH)	8.7	0–45 pg/mL
Cortisol	0.68	6.7–22.6 µg/dL
Follicle-stimulating hormone (FSH)	1.55	4.5–22.5 mIU/mL
Luteinizing hormone (LH)	0.2	19–103 IU/L
Estradiol (E2)	6.7	25–115 pg/mL
Thyroid-stimulating hormone (TSH)	0.9	0.38–5.33 µIU/mL
Free thyroxine (fT4)	0.47	0.61–1.2 ng/dL
Insulin-like growth factor 1 (IGF-1)	117	61–201 µg/L
Prolactin	45	3.3–26.7 ng/mL
Urinary density	1003	1010–1030
Serum osmolality	301	280–295 mosm/kg
Urine osmolality	112	300–1000 mosm/kg

**Fig. 2:**
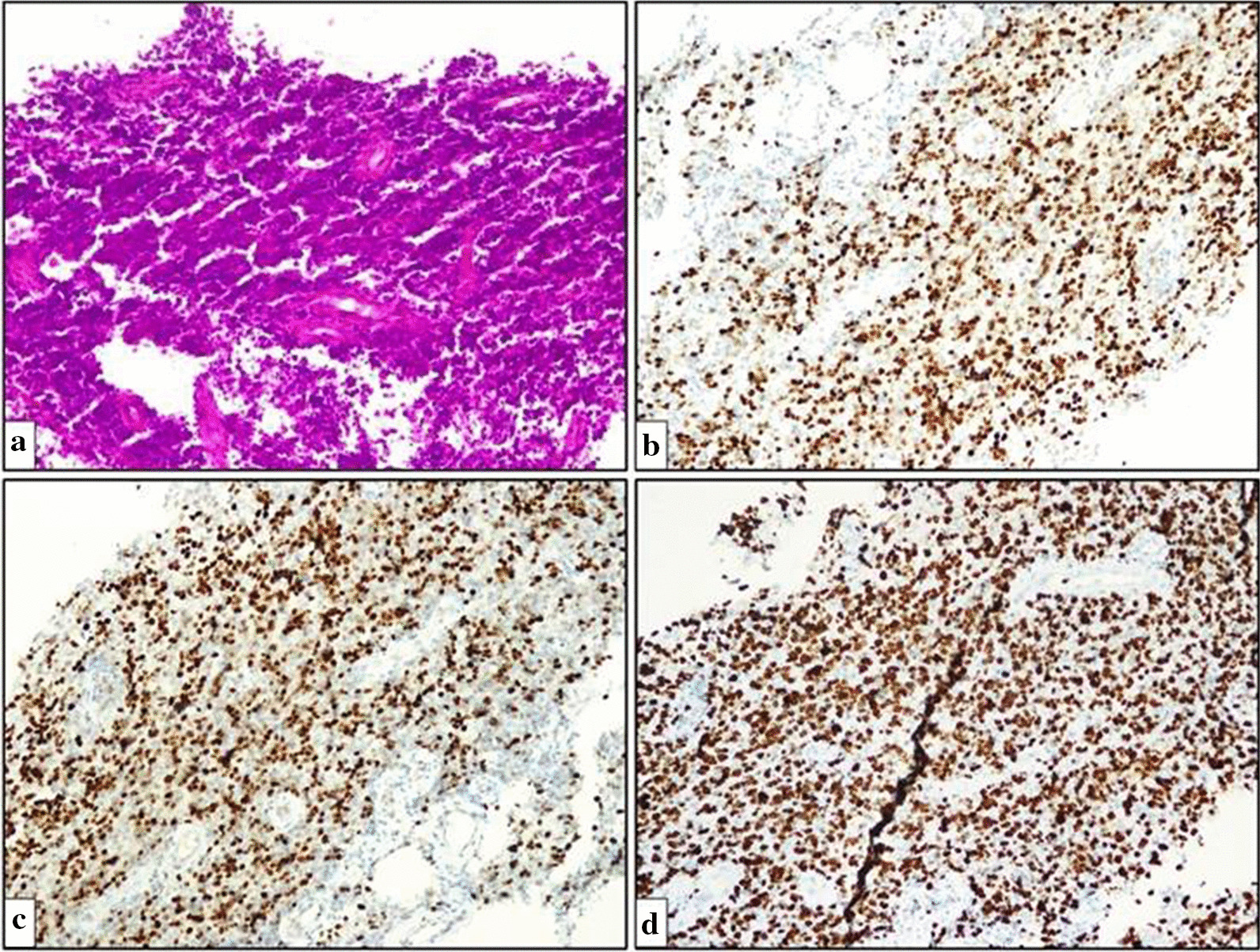
Microscopic image of diffuse large B-cell lymphoma. **a** Diffuse growth pattern with large cells with amphophilic cytoplasm, eccentric nuclei and one central nucleoli. Centroblasts with basophilic cytoplasm, vesicular chromatin and prominent nucleoli close to the nuclear membrane are also observed (hematoxylin and eosin, ×100). **b** Immunohistochemistry-positive neoplastic lymphoid cells for CD20 (×100). **c** Immunohistochemistry-positive neoplastic lymphoid cells for BCL6 (×100). **d** Ki67 immunohistochemistry indicated a high proliferative index of tumor cells, approaching 50% (×100)

The patient was evaluated in the multidisciplinary endocrinology-neurosurgery council, and it was decided to perform a biopsy from the lesion in the hypothalamus for tissue diagnosis. The histopathological features of the surgical tissue biopsy were suggestive of diffuse large B-cell lymphoma (Fig. 2).

The patient was referred to a medical hematologist for further management. She was scheduled for five cycles of an intravenous high-dose methotrexate-based regimen every 14 days (methotrexate 3.5 g/m^2^, vincristine 2 mg/day, calcium folinate 4 × 25 mg/3 days). Unfortunately, after the second cycle of chemotherapy, the patient died due to sepsis.

## Discussion

In this unique case, hypothalamic lymphoma was detected in a patient presenting with acute developing polyuria, polydipsia and mental state changes. Primary hypothalamic lymphoma is a rare condition. In our case, hypopituitarism and DI were caused by the effect of the hypothalamic mass, without pituitary involvement. This case is presented as a rare condition and to show the findings of pituitary insufficiency developing due to mass effect without pituitary involvement.

Primary central nervous system lymphoma (PCNSL) usually presents with focal mass-induced symptoms (56–70%), neuropsychiatric findings (32–43%) and seizures (11–14%) [5]. PCNSL presented with DI and panhypopituitarism due to an isolated hypothalamic involvement is a very rare clinical condition. In our case, benign/malignant tumors, infiltrative lesions and granulomatous diseases were considered in the differential diagnosis. The etiology of the patient’s initial findings was investigated using pituitary function tests, measurement of angiotensin converting enzyme (ACE) level, cranial MRI, and computed tomography (CT) scans of the neck, thorax and abdomen. Biopsy was consistent with the nature of the hypothalamic lesion.

Pituitary macroadenoma, craniopharyngioma, meningioma, metastasis, hamartoma, glioma and granulomatous diseases such as sarcoidosis and tuberculosis are some of lesions which involve the hypothalamus and the third ventricular region [6]. Scans of the chest, abdomen and pelvis were negative for any primary tumor in our patient. Neurosarcoidosis (NS) usually occurs with cranial and peripheral nerve palsy, seizures and congestive disorders. Less commonly, the HP region is affected, with an estimated incidence of 2.5% [7], which may lead to pituitary hormone abnormalities. There was no finding favoring systemic sarcoidosis in the radiological images of our patient. In addition, serum and urinary calcium and ACE levels were normal. However, it has been reported that the serum ACE level is of limited value in establishing the correct diagnosis of NS with HP involvement [8]. Therefore, we could not completely exclude sarcoidosis.

Since hypothalamic PCNSL does not have a specific radiological appearance, diagnosis by radiological methods is very difficult. Currently, the first-line imaging techniques in detecting lesions of PCNSL are MRI and CT. PCNSL is generally demonstrated as a hyperdense lesion on CT due to the hypercellularity. In addition, lymphomas develop homogeneously, which show limited diffusion in diffusion-weighted MRI [9]. In the majority of cases, MRI findings support the diagnosis of PCNSL. However, in some cases with glioblastoma multiforme (GBM), metastatic lesions and non-neoplastic lesions, the diagnosis may be challenging [10]. ^1^H-magnetic resonance spectroscopy may be helpful in the differential diagnosis between PCNSL and GBM [11]. Since PCNSLs do not show an intense vascularization, they show higher regional cerebral blood volume in dynamic sensitivity contrast-enhanced MRI compared to normal brain tissue, but lower than GBMs [12, 13]. Lesions of PCNSL generally show increased uptake of ^18^F-fluorodeoxyglucose (^18^F-FDG). Therefore, ^18^F-FDG positron emission tomography is generally useful in distinguishing hypermetabolic lesions of PCNSL from infection-associated hypometabolic lesions [14]. However, radiological imaging methods are not definitive for a diagnosis for PCNSL, which should be made by histopathological evaluation with stereotactic or open biopsy [15]. In accordance with the literature data, cranial imaging of our patient revealed a lesion suggestive of lymphoma, and the diagnosis was confirmed after histopathological evaluation.

The standard treatment for PCNSL involves two stages: high-dose methotrexate (HD-MTX)-based induction and whole-brain radiotherapy-based consolidation [11]. A recent study recommended the MATRix regimen (combination of HD-MTX, high-dose cytarabine [HD-AraC] thiotepa and rituximab) as standard chemo-immunotherapy [16]. New agents such as lenalidomide and ibrutinib offer successful results in relapse cases [11]. Programmed cell death protein 1 (PD-1)/programmed death-ligand 1 (PD-L1) expression was detected in 42% of PCNSL cases, and they show promise in these patients [17]. A standard high-dose methotrexate-based regimen was started in our patient. However, the response could not be evaluated because the patient died due to secondary causes.

The occurrence of hypothalamic lesions with findings mimicking pituitary apoplexy is an extremely rare condition [18]. The most common symptoms and findings of hypothalamic lesions are DI, hypernatremia, hyper- or hypothermia, and eating, sleep and behavioral disorders [19]. Our patient initially presented with altered circadian sleep rhythms and then subsequently with polydipsia, polyuria, extremely diluted urine and elevated serum sodium indicating DI. The standard validation method for DI is the water restriction test [20], which could not be performed due to the impaired clinical status of our patient. The diagnosis of central DI was indirectly confirmed by the patient’s good response to the synthetic antidiuretic hormone (ADH) analog desmopressin.

"Empty sella" is an anatomical and radiological definition in which the subarachnoid space is herniated to the sella turcica and the pituitary gland is reduced [21]. Cerebral tumors or idiopathic intracranial hypertension can lead to the herniation of the subarachnoid space through increased intracranial pressure, which causes the compression of the pituitary gland. Other causes of empty sella syndrome include pituitary adenoma, sheehan syndrome, radiotherapy, trauma and surgery. The prevalence of hypopituitarism in empty sella syndrome is 52%, while the prevalence of isolated pituitary hormone insufficiency is 21%, and multiple pituitary axis dysfunction 30% [22]. The onset of pituitary insufficiency usually progresses gradually. At first, growth hormone (GH) and gonadotropin hormone are impaired, followed by decreased thyroid-stimulating hormone (TSH), adrenocorticotropic hormone (ACTH) and prolactin secretion [23]. It remains unknown whether the hormonal dysfunction develops in this order in empty sella syndrome. Low insulin-like growth factor 1 (IGF-1) concentration is usually the first sign of hypopituitarism, but a normal IGF-1 level alone cannot exclude the presence of other hormonal abnormalities [24]. The growth hormone axis was preserved in our patient at initial admission, while all adrenal, gonadal and thyroidal axes were disturbed. Somatotropic cells are concentrated in the periphery of the pituitary gland [25]. In our patient, the height of the pituitary gland was found to be decreased significantly, especially in the central region, and the growth hormone axis may not have been affected due to partially preserved peripheral area.

The vast majority of people with empty sella syndrome do not show any symptoms, usually have no hormonal abnormalities and do not need treatment. In empty sella syndrome, hormone replacement therapy should be evaluated for each hormone and started in the appropriate order. In the presence of multiple hormone deficiencies, it is recommended that patients start replacement therapy with hydrocortisone followed by levothyroxine. Sex hormone replacement should be initiated when the patient's condition stabilizes [21]. In our patient, after the pituitary function was evaluated, replacement therapy was started with hydrocortisone, and then levothyroxine treatment was added.

Finally, our results suggested a partial impairment of anterior pituitary function and a total loss of posterior pituitary function. Based on MRI findings, there was no infiltration of the pituitary gland; thus, the hormonal disturbances were attributed to the infiltrative lesion in the hypothalamic region.

## Conclusion

PCNSL complicated by hypopituitarism is a rare condition; however, it attracts attention due to the lack of specific clinical findings and the possibility of misdiagnosis due to pituitary adenoma or many other causes. Since the differential diagnosis of primary hypothalamic lesions is extensive, this case highlights the difficulty in making a diagnosis. In patients with sellar or parasellar masses accompanied by pituitary dysfunction, the presence of a CNS lymphoma should be evaluated. As described in our case report, an urgent biopsy may be life-saving when laboratory and imaging methods are not sufficient for a definitive diagnosis. Appropriate timing of treatment is vital in hypopituitarism.

## Data Availability

All of the data and materials will be available from the corresponding author upon request.

## Abbreviations

CNS: Central nervous system
DI: Diabetes insipidus
MRI: Magnetic resonance imaging
ACE: Angiotensin-converting enzyme
CT: Computed tomography
NS: Neurosarcoidosis
HP: Hypothalamic-pituitary
PCNSL: Primary central nervous system lymphoma
^18^F-FDG: ^18^F-fluorodeoxyglucose
